# Galectins in Protozoan Parasitic Diseases: Potential Applications in Diagnostics and Therapeutics

**DOI:** 10.3390/cells12232671

**Published:** 2023-11-21

**Authors:** Cássio Meira, Jaqueline Silva, Helenita Quadros, Laís Silva, Breno Barreto, Vinícius Rocha, Larissa Bomfim, Emanuelle Santos, Milena Soares

**Affiliations:** 1 Gonçalo Moniz Institute, Oswaldo Cruz Foundation (FIOCRUZ), Salvador 21040-900, Bahia, Brazil; jaquelinewang.sw@gmail.com (J.S.); helenita_quadros@hotmail.com (H.Q.); laisperez1@hotmail.com (L.S.); brenoc.barreto@hotmail.com (B.B.); vinicius.rocha@fieb.org.br (V.R.); larissambomfim@gmail.com (L.B.); 2 SENAI Institute of Innovation in Health Advanced Systems (ISI SAS), University Center SENAI/CIMATEC, Salvador 41650-010, Bahia, Brazil; emanuelle.santos@fieb.org.br; 3 Institute of Health Sciences, Federal University of Bahia (UFBA), Salvador 40170-110, Bahia, Brazil

**Keywords:** galectins, Chagas disease, leishmaniasis, malaria

## Abstract

Neglected tropical diseases (NTDs) constitute a group of diseases that generally develop in tropical or subtropical climatic conditions and are related to poverty. Within the spectrum of NTDs, diseases caused by protozoa such as malaria, Chagas disease, and leishmaniasis exhibit elevated mortality rates, thereby constituting a substantial public health concern. Beyond their protozoan etiology, these NTDs share other similarities, such as the challenge of control and the lack of affordable, safe, and effective drugs. In view of the above, the need to explore novel diagnostic predictors and therapeutic targets for the treatment of these parasitic diseases is evident. In this context, galectins are attractive because they are a set of lectins bound to β-galactosides that play key roles in a variety of cellular processes, including host-parasite interaction such as adhesion and entry of parasites into the host cells, and participate in antiparasitic immunity in either a stimulatory or inhibitory manner, especially the galectins-1, -2, -3, and -9. These functions bestow upon galectins significant therapeutic prospects in the context of managing and diagnosing NTDs. Thus, the present review aims to elucidate the potential role of galectins in the diagnosis and treatment of malaria, leishmaniasis, and Chagas disease.

## 1. Introduction

Neglected tropical diseases (NTDs) encompass a group of diseases that are caused by a diverse array of transmissible microorganisms such as viruses, fungi, parasites, and bacteria. These maladies typically manifest within tropical or subtropical climatic regions and are intrinsically intertwined with the issue of poverty [1]. According to the World Health Organization (WHO), NTDs put the lives of more than 200 million people at risk, in addition to having serious health, social, and economic consequences for more than a billion people who are in a situation of poverty and vulnerability worldwide [2]. Notably, within the spectrum of NTDs, protozoa-induced afflictions such as malaria, Chagas disease, and leishmaniasis exhibit the highest mortality rates, contributing to approximately 234 million reported cases and more than 400,000 deaths worldwide, making them a major public health problem [3,4].

Malaria is a protozoan disease whose etiological agents are parasites of the genus *Plasmodium*, responsible for about 2000 daily deaths worldwide [5]. Among the existing species of *Plasmodium*, it is known that five of them are capable of infecting humans: *P. vivax, P. falciparum, P. ovale, P. malariae,* and *P. knowlesi*; however, it is noteworthy that the majority of mortalities resulting from malaria can be attributed to *P. falciparum* [5,6]. In 2021, the WHO documented approximately 247 million cases of malaria, resulting in 619,000 deaths worldwide. A substantial majority of these deaths occur among children under the age of five who reside in sub-Saharan Africa [6,7,8]. The diagnostic and parasitemia classification of malaria is a complex undertaking, and without comprehensive and dependable knowledge, the incidence of malaria-induced deaths is poised to escalate [9]. Presently, the principal strategies employed in combating malaria transmission revolve around the utilization of artemisinin-based combination therapies (ACT) in conjunction with the deployment of insecticide-treated bed nets [10]. However, it is imperative to acknowledge the existence of parasites that exhibit resistance to artemisinin, thereby fostering the persistence of the parasite post-treatment [5].

Another protozoan disease is Chagas disease, which is caused by *Trypanosoma cruzi* and transmitted to humans via contact with the excrement of blood-feeding triatomine insects, rendering its transmission primarily vector-borne [11]. Nevertheless, this parasite can also be disseminated through non-vector means, including congenital transmission, blood transfusion or organ transplantation, contaminated food or beverages, and accidental laboratory exposure [12]. The WHO estimates that 6 to 7 million people worldwide will be infected with *T. cruzi* in 2023, predominantly in Latin American countries where Chagas disease is endemic. However, there are cases of Chagas disease in other countries around the world, proving that the disease crossed borders and became a global epidemic [13]. Currently, the main strategy for Chagas disease prevention in endemic regions revolves around vector control, executed through the indoor application of pyrethroids and enhancements in housing conditions [14]. Regrettably, there has been limited investment in the development of vaccines and pharmaceuticals for Chagas treatment. Furthermore, a substantial number of patients in endemic areas confront impediments to accessing diagnostic tests. Additionally, no methodologies presently exist for assessing the efficacy of treatment regimens and monitoring disease progression [15].

Leishmaniasis, which is a multifaceted group of diseases caused by 20 species of protozoa of the genus *Leishmania*, is transmitted anthropologically or zoonotically through the bite of sandflies [16]. Individuals affected by Leishmaniasis often manifest a spectrum of symptoms, including irregular fever, anemia, atrophy, hepatosplenomegaly, and low immunity [17]. There are three main clinical forms: cutaneous, visceral (lethal form), and mucocutaneous Leishmaniasis, which depend on the interaction between the host’s immune system and the specific *Leishmania* species involved [18]. The various clinical presentations of Leishmaniasis are distributed globally and are endemic diseases in approximately 100 countries, affecting 12 million individuals and putting 350 million at risk of infection [19]. Furthermore, it is worth noting that the World Health Organization (WHO) estimates that over 30,000 new cases of visceral Leishmaniasis and more than one million cases of cutaneous Leishmaniasis emerge annually [16]. A significant contemporary challenge in the diagnosis of Leishmaniasis resides in the limited accessibility of diagnostic methods, which are predominantly restricted to reference hospitals and advanced laboratories. These techniques are often capable of detecting only one *Leishmania* species at a time. Consequently, there is a need to simplify the diagnosis in endemic regions [20].

In addition to being caused by protozoa, these NTDs share commonalities such as the difficulty of being controlled and the lack of affordable, safe, and effective pharmaceutical interventions [21]. In the case of malaria, there is a problem of resistance of the parasite to antimalarial drugs [22]. In 1957, the first case of chloroquine-resistant malaria was reported, and then the use of mefloquine and sulfadoxine-pyrimethamine was adopted; however, in the 1980s, resistance against these drugs was also reported [23]. Presently, the most efficacious approach hinges on combination therapy incorporating artemisinin. However, WHO advocates against its monotherapy application, favoring a dual-drug regimen characterized by distinct mechanisms of action to curtail parasite proliferation within the infected host [24]. Thus, the need for new, effective therapies to combat malaria is evident.

During the acute, congenital, and reactivation phases of Chagas disease, therapeutic interventions involve the administration of benznidazole and nifurtimox. These pharmacological agents exhibit variable efficacy, demonstrating disease suppression rates ranging from 50% to 80%, whereas in asymptomatic chronic infections, their effectiveness is approximately 20% to 60% [25]. Benznidazole and nifurtimox belong to the nitroheterocyclic drug class and exert their therapeutic influence by disrupting essential biological processes critical for the parasite’s survival. It is noteworthy that the administration of these drugs is linked to a spectrum of adverse events [26]. Furthermore, the efficacy of benznidazole and nifurtimox is notably diminished in elderly individuals and during the chronic phase of the disease. These medications should be administered with caution, and they are contraindicated for pregnant women, individuals with renal insufficiency, and those afflicted with hepatic disorders. Moreover, they are known to induce a range of undesirable side effects, including myalgia, pruritus, dermatological eruptions, headaches, and gastrointestinal discomfort [13,26]. Concurrently, the therapeutic approach for Leishmaniasis entails the utilization of chemotherapy involving antimonials for the cutaneous and mucocutaneous manifestations, while amphotericin B is employed for the treatment of the visceral form of the disease [27]. Nevertheless, these pharmaceutical agents frequently elicit adverse effects, exhibit suboptimal efficacy, are associated with toxicities, incur substantial financial burdens, and are plagued by the emergence of drug-resistant strains, leading to patient relapses [17]. Therefore, the development of new effective drugs for the treatment of Chagas disease and Leishmaniasis is still crucial.

In light of the aforementioned considerations, the need to explore novel diagnostic indicators and therapeutic targets for the treatment of these parasitic diseases is evident. In this sense, galectins emerge as a promising avenue because they are a set of lectins bound to β-galactosides with fundamental roles in the regulation of cellular functions such as differentiation, angiogenesis, cell growth, and host defense mechanisms [28]. In addition, galectins are widely expressed by several cell types, such as macrophages, monocytes, and epithelial cells; therefore, the evaluation of serum levels of galectin may be useful as diagnostic markers of parasitic diseases [29]. In parasitic infections, galectins engage with the glycoconjugates present on the parasite’s surface, thereby facilitating recognition, the initiation of innate immune responses, and antigen processing. These functions bestow upon galectins significant therapeutic prospects in the context of managing and diagnosing NTDs [30]. Furthermore, it is noteworthy that galectins have been associated with numerous parasitic pathogens, including those under scrutiny in this review [31]. Consequently, the present review aims to elucidate the pivotal role of galectins in the diagnosis and therapeutic intervention of malaria, Leishmaniasis, and Chagas disease.

## 2. Galectins

**Figure 1:**
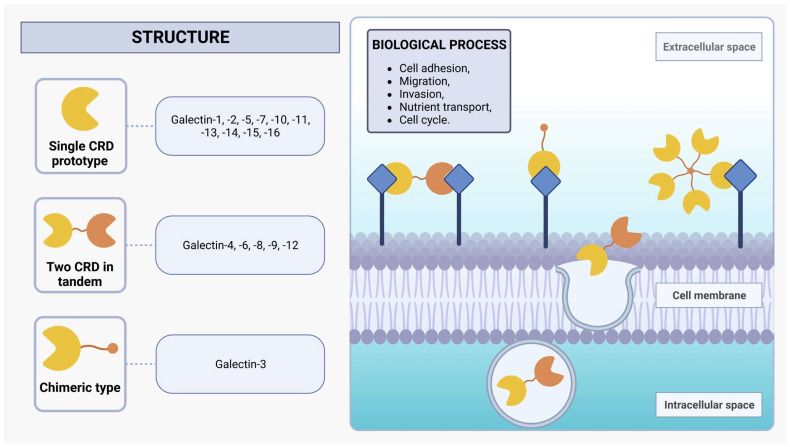
Structure of galectins. Mammalian galectins are classified into three subclasses based on their structure: single CRD, two CRDs in tandem, and chimeric type. They form dynamic lattice structures by interacting with cell-surface glycoprotein receptors, influencing diverse biological processes such as adhesion, migration, invasion, nutrient transport, and cell cycle regulation.

Galectins are a protein family of *N*-acetyllactosamine-containing glycan-binding lectins that are expressed in many cell types, including monocytes, macrophages, dendritic cells (DCs), mast cells, B cells, and T cells [29,30]. From worms to humans, galectins are widespread throughout the animal kingdom and also expressed in protozoan parasites such as *L. major* [30]. Initially recognized for their capacity to bind to β-galactoside sites, galectins employ one or two evolutionarily conserved carbohydrate recognition domains (CRDs), each composed of approximately 130 amino acids [32,33]. Within mammals, the galectin family encompasses 16 distinct proteins, which can be categorized into three subgroups based on their structural characteristics: single CRD prototypes (galectin-1, -2, -5, -7, -10, -11, -13, -14, -15, and -16), those with two CRDs arranged in tandem (galectin-4, -6, -8, -9, and -12), and the chimeric type (galectin-3), characterized by a single CRD in conjunction with an extended *N*-terminal domain rich in proline and glycine (Figure 1) [30,33,34,35]. Moreover, investigations into non-mammalian galectins, particularly those derived from marine sponges, have unveiled novel functionalities associated with the formation of tetrameric galectin arrangements [36,37,38]. The alteration in protein structure assembly changes its activity profile, such as stability and ligand specificity [37,39].

Galectins exhibit functionalities in both extracellular and intracellular environments. These versatile proteins engage in diverse biological processes, including protein-protein interactions and nuclear translocation, wherein they play a pivotal role in enhancing the stability of DNA-protein interactions [28]. Additionally, galectins can also be non-classically secreted via an exosome-mediated route, facilitating the formation of a dynamic lattice structure via binding with cell-surface glycoprotein receptors [32,40]. The interplay between transmembrane glycoproteins and the dynamic galectin lattice is proportional to the number of branches of their *N*-glycans and the Golgi complex glycosyltransferase activities that generate several β-galactoside binding epitopes present on the glycoproteins [29,32]. These glycoproteins are central to the biological effects of galectins, and the interactions between them orchestrate an array of pivotal cellular activities, encompassing cell adhesion, migration, invasion, and nutrient transport, as well as the regulation of cell cycle dynamics, including growth, proliferation, and apoptosis [32,41]. The impact of galectins on cellular functions emphasizes their critical involvement in the orchestration of immune system regulation and inflammatory responses by modulating signaling pathways and cell behavior under normal physiological conditions and in the context of pathological states [42,43].

The participation of galectins in various pathological conditions has been substantiated by multiple investigations, thereby underscoring their potential utility as targets for innovative therapeutic approaches, diagnostic methodologies, or predictive biomarkers. This potential is particularly attractive for the NTDs addressed in this review, especially with regard to the development of new therapeutic approaches, since there is a lack of effective medicines to treat patients with malaria, Chagas disease, or leishmaniasis [22,23,24,25,26,27]. Furthermore, the molecular targets traditionally employed in the process of developing novel drugs, such as cruzain, sterol 14 α-demethylase, trypanothione reductase, and falcipain, have thus far failed to yield new therapeutic options for patients. Consequently, galectins emerge as promising and viable targets for the development of new drugs [44,45,46]. Additionally, the utility of galectins extends beyond therapeutic applications, presenting themselves as valuable candidates for the development of diagnostic methodologies or as biomarkers indicative of disease progression [28,30].

For instance, galectin-3, a potent proinflammatory protein, has been identified as a significant biomarker for cardiovascular disorders such as atherosclerosis and autoimmune myocarditis [47]. Galectin-3 expression may precipitate macrocalcification, thereby increasing the risk of heart failure [48]. In contrast, galectin-9 tends to evoke an anti-inflammatory response, rendering it a promising intervention in the treatment of autoimmune and inflammatory conditions such as intracerebral hemorrhage [49], cancer [50], allograft rejection [51], and chronic asthma [52]. Galectin-16, expressed in the placenta, plays a central role in the maternal-fetal interface, promoting immune tolerance through T-cell apoptosis and trophoblast cell upregulation. Moreover, it is associated with the pathogenesis of preeclampsia disorders [53,54]. It is also noteworthy that galectins have been implicated in pivotal roles concerning central and peripheral tolerance mechanisms, as well as in the orchestration of processes and defense pathways against pathogenic agents [28,32].

In this scenario, parasitic glycoconjugates assume a pivotal role in the invasion of host cells, as they not only facilitate pathogen recognition but also elicit effector responses through interactions with host galectins [30]. Several mechanisms come into play, including the prevention of pathogen attachment to the host cell surface (galectin-1 and galectin-9), direct pathogen neutralization (galectin-4 and galectin-8), or promotion of pathogen clearance via phagocytosis (galectin-3) [55]. Among the galectins identified so far, galectin-3 (Gal-3) has been implicated in various parasitic infections, such as malaria [56], *Leishmania* [57], *Trypanosoma cruzi*, *Toxoplasma gondii* [58], and *Schistosoma mansoni* [59]. This singular chimeric galectin modulates the immune response with specific pleiotropic functions; nevertheless, its effects are contingent upon its presence in distinct cell types and can manifest in activated T lymphocytes, B lymphocytes, populations of monocytes and macrophages, neutrophils, mast cells, and eosinophils [30,40,42].

## 3. The Role of Galectins in *Plasmodium* Infection

**Figure 2:**
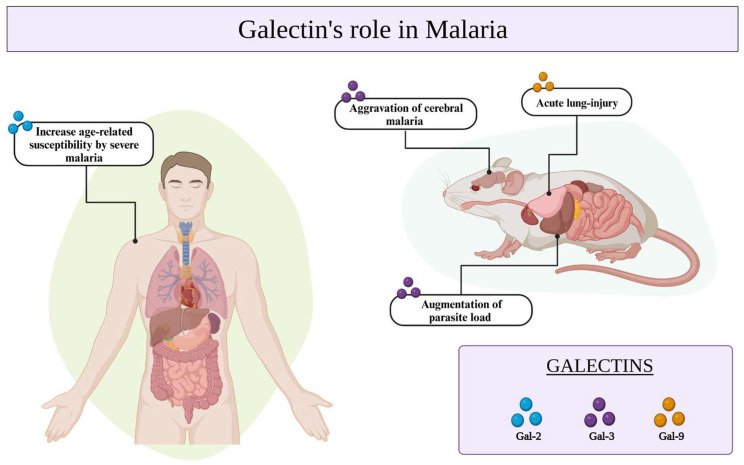
Galectins-2, -3, and -9 play crucial roles in *Plasmodium* infection, contributing to the development of severe malaria in aging populations, the exacerbation of cerebral malaria, and acute lung injury. Additionally, it is a protective molecule that regulates parasite load in *P. yoelii* infections.

Galectins have been implicated in numerous immunological processes as well as in the recognition of pathogens via specific interactions with glycosylated receptors on the surfaces of host cells or microorganisms [56]. In the context of malaria, the galectins seem to be involved in controlling *Plasmodium* infection and have been associated with the severity of malaria [60], particularly in cases of cerebral malaria (CM) exacerbation [61] (Table 1). The interaction between Gal-9 and its receptors holds significant importance in murine malaria-associated acute lung injury [61]. Additionally, the presence of Gal-2 has been associated with an increased susceptibility to severe malaria in age-related populations [62]. Gal-3, which is predominantly expressed in macrophages, has the potential to alter the pathogenic course of experimental CM through its binding to the endogenous oligosaccharides on matrix proteins and its release after lysis of brain-infiltrating macrophages (Figure 2) [62,63].

In a study developed by Toscano and colleagues [56], an investigation was undertaken to evaluate the parasite load in mice deficient in Gal-3 or Lgals3 knockout mice infected with *Plasmodium yoelii*, *Plasmodium berghei*, and *Plasmodium chabaudi*. Interestingly, only upon *P. yoelii* infection did the Gal-3 knockout mice exhibit a significant reduction in parasitemia compared to wild-type (WT) mice. This outcome suggests that the presence of Gal-3 is associated with an augmentation of parasite load, specifically in the case of *P. yoelii* infection. Conversely, in the cases of *P. berghei* and *P. chabaudi* infections, the parasitemia levels observed in the knockout mice were similar to those in WT mice. This finding suggests that Gal-3 specifically stimulates *P. yoelii* replication or infectivity [56].

In the same investigation, Toscano and colleagues [56] assessed the immune response against *P. yoelii* in Lgals3^−/−^ (galectin-3-deficient) and WT mice by measuring IgG antibody titers against *P. yoelii* merozoite surface protein 1_19_ (PyMSP1_19_), a well-recognized vaccine candidate antigen [64]. Remarkably, their findings revealed higher levels of anti-MSP1_19_ IgG2b antibodies in Lgals3^−/−^ mice compared to WT mice. This observation reinforces the involvement of Gal-3 in the replication of *P. yoelii*, as the elevated antibody response suggests a more robust immune reaction against the parasite in the absence of Gal-3 [56]. Gal-9 is another galectin recognized as an immune modulator capable of inducing cell death by interacting with T cell immunoglobulin and mucin domain-3 (Tim-3). This interaction promotes the inhibition of various pro-inflammatory cytokines, including TNF, IL-6, and IL-1α, while simultaneously stimulating the production of IL-10 [65]. Based on this, studies have reported immunoregulatory activities of Gal-9 in animals and patients with malaria, highlighting increased Gal-9 and Tim-3 expression in different stages of the *Plasmodium* infection and, hence, an increase in malaria severity [66,67,68,69].

Dembele and colleagues [66] assessed the levels of Gal-9 in plasma samples obtained from individuals from Thailand with acute malaria caused by *P. falciparum*, including nine cases of severe malaria and 41 cases of uncomplicated malaria, over different time points (days 0, 7, and 28). Their findings revealed that Gal-9 levels were significantly elevated on day 0 compared to the other days assessed, and these levels were higher in individuals with severe malaria as opposed to those with uncomplicated cases on both day 0 and day 7. Consequently, this study indicates that Gal-9 levels in the blood plasma of malaria patients are markedly higher in cases of severe malaria compared to uncomplicated cases, potentially serving as a biomarker for disease severity. Furthermore, in both types of malaria (severe and uncomplicated), Gal-9 levels were associated with various pro- and anti-inflammatory cytokines and chemokines, including TNF, IL-6, IFN-α2, IFN-γ, IL-1Ra, and IL-10. These associations peaked at day 0 but disappeared by day 28, suggesting that the high levels of Gal-9 released during acute malaria may play a role in terminating the immune response by binding to Tim-3 and that the dynamics in Gal-9 levels are reflective of the severity of malaria over time [28,66].

Xiao and colleagues investigated the roles of Tim-3 and its ligand Gal-9 in the development of liver injury during malaria using a murine model infected with the *Plasmodium berghei* ANKA strain [67]. This study detected a substantial increase in Tim-3 and Gal-9 mRNA expression in the livers and spleens of *P. berghei* ANKA-infected mice over time. This upregulation of Tim-3 and Gal-9 expression correlated with the overexpression of both pro-inflammatory cytokines (IL-1β, IL-6, and TNF-α) and anti-inflammatory cytokines (IL-10) in the liver. Additionally, IL-4, IL-6, and IL-10 were overexpressed in the spleen following infection. These findings collectively indicate that the Tim-3/Gal-9 pathway plays a crucial role as a key regulator in the inflammatory pathways within the liver, leading to liver injury as the malaria infection progresses [67,68].

Recently, Bailly classified the Tim-3 receptor as an important checkpoint due to its induction by parasites, particularly *P. falciparum* or *P. vivax*, as a strategy to evade the host immune system [70]. Notably, the expression of Tim-3 is upregulated during infection, particularly in cases of acute malaria, and concomitantly, Gal-9 levels also increase. These elevated levels of both Tim-3 and Gal-9 were found to correlate with the severity of the disease [59]. As a result, the upregulation of Tim-3 and Gal-9 during malaria infection can lead to their overexpression, which is associated with tissue damage, particularly in the liver and lungs [70,71].

With the aim of investigating more extensively the role of the Gal-9/Tim-3 pathway, Liu et al. and Wu et al. performed studies to assess the impact of blocking Gal-9 and Tim-3 interactions by treatment with alpha (α)-lactose on liver immunopathology during the erythrocytic stage of malaria in a *P. berghei* ANKA-infected mouse model [61,69]. Treatment with alpha (α)-lactose reduced host survival rates and increased peripheral blood parasitemia. However, an unexpected outcome emerged as the pro-inflammatory cytokine levels in the lungs and liver were more pronounced in the alpha (α)-lactose-treated group compared to control-infected mice. This suggests that the blockade of galectin-receptor interactions by α-lactose exacerbates the inflammatory responses in the liver and lungs during *P. berghei* infection [61,69,72]. Additionally, a study developed by Duan et al. showed that Gal-9 is also involved in the aggregation of *P. berghei*-infected red blood cells, which is crucial for blood–brain barrier injuries and, hence, in the aggravation of CM [73]. Taken together, these results support the hypothesis that, similar to other galectins, Gal-9 also plays a role in influencing the severity of malaria [73,74]. In light of these findings, the galectins, including Gal-9, may indeed be considered potential targets for the development of antimalarial therapies.

**Table 1:** Main effects of galectins on Malaria disease experimental models.

Reference	Experimental Model	Species	Main Results
[61]	Mice	*P. berghei* ANKA strain	Treatment with alpha (α)-lactose reduced host survival rates and increased peripheral blood parasitemia. However, an unexpected outcome emerged as the pro-inflammatory cytokine levels in the lungs and liver were more pronounced in the alpha (α)-lactose-treated group compared to control-infected mice. This suggests that the blockade of galectin-receptor interactions by α-lactose exacerbates the inflammatory responses in the liver and lungs during *P. berghei* infection.
[62]	Human	*P. falciparum* and *P. vivax*	Gal-2 has been associated with an increased susceptibility to severe malaria in age-related populations.
[63]	Mice	*P. falciparum* and *P. vivax*	Gal-3 has the potential to alter the pathogenic course of experimental cerebral malaria (CM) through its binding to the endogenous oligosaccharides on matrix proteins and its release after the lysis of brain-infiltrating macrophages.
[56]	Mice	*P. yoelii*, *P. berghei* and *P. chabaudi*	Upon *P. yoelii* infection, the Gal-3 knockout mice exhibited a significant reduction in parasitemia compared to wild-type (WT) mice; however, in the cases of *P. berghei* and *P. chabaudi* infections, the parasitemia levels observed in the knockout mice were similar to those in WT mice. This finding suggests that Gal-3 stimulates specifically *P. yoelii* replication or infectivity. Also, additional experiments with *P. yoelii* revealed that a more robust immune reaction against the parasite occurs in the absence of Gal-3.
[66]	Human	*P. falciparum*	Gal-9 levels in the blood plasma of malaria patients were markedly higher in cases of severe malaria compared to uncomplicated cases, potentially serving as a biomarker for disease severity. Additionally, in both severe and uncomplicated malaria, Gal-9 levels were associated with various pro- and anti-inflammatory cytokines and chemokines, including TNF, IL-6, IFN-α2, IFN-γ, IL-1Ra, and IL-10.
[67]	Mice	*P. berghei* ANKA *strain*	The Tim-3/Gal-9 pathway plays a crucial role as a key regulator in the inflammatory pathways within the liver, leading to liver injury as the malaria infection progresses.
[70]	Mice	*P. berghei*	The upregulation of Tim-3 and Gal-9 during malaria infection can lead to their overexpression, which is associated with the severity of malaria and tissue damage, particularly in the liver and lungs.
[72]	Mice	*P. berghei* ANKA *strain*	Gal-9 is also involved in the aggregation of *P. berghei*-infected red blood cells, which is crucial for blood–brain barrier injuries and, hence, in the aggravation of CM.

Cerebral malaria (CM); Galectins (Gal); interferon alpha-2 (IFN-α2); Interferon-gamma (IFN-γ); interleukins (IL); T cell immunoglobulin and mucin domain-containing protein 3 (Tim-3); Tumor Necrosis Factor Alpha (TNF); Wild-Type (WT).

## 4. The Role of Galectins in Leishmaniasis

**Figure 3:**
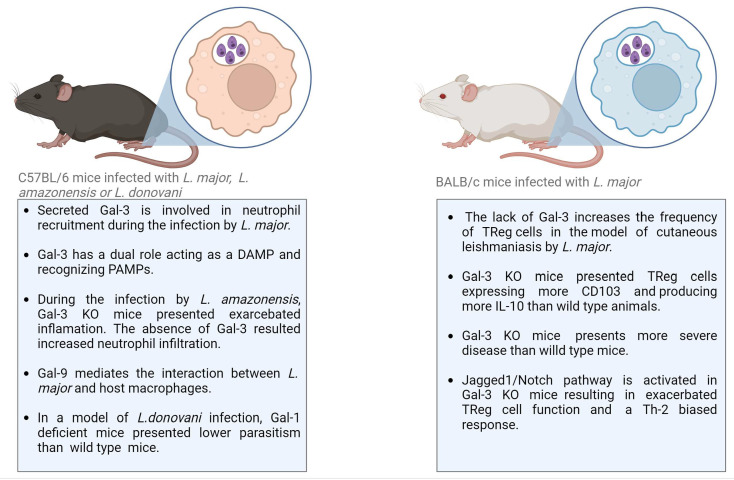
Main findings regarding the roles of Galectins during experimental models of Leishmaniasis. Gal-galectin; DAMP, damage-associated molecular pattern; PAMP, pathogen-associated molecular pattern; TReg, regulatory T-cell; KO, knockout; IL, interleukin; Th, helper T-cell.

In the context of Leishmaniasis, Gal-1 and Gal-3 are the most explored galectins (Table 2; Figure 3). Gal-1, a dimeric galectin, played an important role in the experimental model of visceral Leishmaniasis caused by *L. donovani* infection in C57BL/6 mice. Gal-1 was expressed by antigen-presenting cells and by Th1 lymphocytes in the liver and spleen of infected mice [75,76]. Interestingly, when Gal-1 knockout mice were subjected to infection, they manifested an elevated frequency of IFNγ-producing CD4^+^ T cells in comparison to their wild-type counterparts. Because Gal-1 is associated with the production of IL-10, a cytokine involved in the suppression of anti-parasitic immune responses in infected animals, the levels of IL-10 were also monitored in Gal-1 KO mice [75,76]. No difference in IL-10 production by CD4^+^ Foxp3^+^ or CD4^+^ Foxp3^−^ T cells was found comparing Gal-1 KO and wild-type mice. However, parasitism in Gal-1-deficient mice infected with *L. donovani* was lower than in wild-type mice [76].

In a related context, Gal-3 has also been recognized for its modulatory role in *Leishmania* infection. Gal-3 modulates *Leishmania* infection by binding directly to the parasite or through the modulation of immune cells, such as neutrophils, monocytes, macrophages, and dendritic cells, exerting effects in both innate and adaptive immune responses. The modulation of immune cells is performed by extracellular Gal-3 binding to a receptor on the cell surface in an autocrine or paracrine manner. Gal-3 also acts as a modulator of intracellular proteins [77]. To determine the role of secreted or intracellular Gal-3, exogenous molecules have been used to treat Gal-3-deficient mice or specific cell types. [77]. In a mouse model of cutaneous Leishmaniasis caused by infection with *L. major*, secreted Gal-3 was shown to exert an important function in neutrophil attraction. Gal-3 deficiency resulted in reduced neutrophil infiltration, which was reversed by adding exogenous Gal-3 [78]. This effect was observed despite Gal-3 having shown the opposite effect regarding acting as a chemoattractant for neutrophils in vitro [79]. Thus, the hypothesis that Gal-3 may act as a damage-associated molecular pattern (DAMP) during *Leishmania* infection was raised. Gal-3 is produced and stored in the cytoplasm, but in the presence of a pathogen or prolonged infection, intracellular Gal-3 is released by dying cells or actively by inflammatory cells. Extracellular Gal-3 binds to the pathogen-associated molecular pattern (PAMP) in *Leishmania* [80]. Gal-3 recognizes non-self glycan on the parasite surface; it is considered a pattern recognition receptor (PRR). However, released Gal-3 is found in lesions associated with pathogen infection and modulates the innate immune response, being also considered a DAMP [81,82].

Gal-3 actively modulates the adaptive immune response, regulating the frequency and function of CD4^+^ CD25^+^ Foxp3^+^ T regulatory (TReg) cells. The lack of Gal-3 increases the frequency of TReg cells in the site of infection, as well as in the draining lymph-nodes, in a mouse model of cutaneous Leishmaniasis caused by *L. major* infection. These results correlated with the more severe disease developed in Gal-3 knockout (KO) mice compared to wild-type mice. Gal-3 KO mice also presented TReg cells expressing CD103 and producing more IL-10 than wild-type animals, suggesting a greater tissue-specific trafficking, recruitment, and modulatory microenvironment in the absence of Gal-3 [83]. Notch signaling was also altered in Gal-3 KO mice and may contribute to the regulatory phenotype and the course of *L. major* infection. Fermino and colleagues [83] demonstrated Jagged1/Notch activation in Gal-3 KO mice infected by *L. major*, resulting in exacerbated TReg cell function. Notch signaling pathways are involved in T-cell development in central and peripheral lymphoid organs, as well as in the naïve T-cell differentiation into T-helper (Th) 1 or 2 phenotypes, being the Jagged ligand associated signaling related to Th-2 biased response [84,85]. Endogenous Gal-3 is involved in the regulation of Jagged1/Notch and the control of TReg cell development. The absence of Gal-3 results in the high expression of Jagged1 and Notch’s targeted gene Hes-1 by T_Reg_ cells and bone-marrow-derived dendritic cells. These data showed that components of the Notch signaling pathway are altered in the Gal-3 KO animals, and this lectin exerts immunoregulatory activity through TReg-cell development, thus contributing to disease severity in the mouse model of cutaneous Leishmaniasis [83,86].

Gal-3 has been described as a PRR able to bind lipophosphoglycan (LPG), the major surface molecule found in *Leishmania* parasites [80]. Since the glycan structure changes among the species, epitopes recognized by Gal-3 may be altered. Determining the binding to different epitopes in LPG is important to understand the host-parasite interaction and species-specific tissue tropism observed in different clinical forms of *Leishmaniases*. Gal-3 recognition of LPG in the surface *L. major* results in the cleavage of the lectin by the parasite’s zinc metalloproteases [80]. The same study showed that Gal-3 did not recognize *L. donovani* LPG, suggesting the species-specificity of LPG-binding by Gal-3 and its activity as PRR [80]. Gal-9 can also recognize *L. major* through its interaction with specific epitopes. Even presenting these similarities regarding parasite recognition, according to Pelletier and colleagues, only Gal-9 can mediate the interaction between *L. major* and host macrophages [57]. Based on the reported role of Gal-3, the action of *Leishmania* metalloproteases may abrogate the innate immunomodulation of Gal-3 and may contribute to disease pathogenesis [80]. Indeed, Gal-3 has a critical role during *Leishmania* infection. During the experimental infection of mice with *L. amazonensis*, Gal-3 was involved in the control of parasite invasion, replication, recruitment of leukocytes, and the biogenesis of endocytic vesicles [87]. The absence of Gal-3 in KO mice resulted in exacerbated inflammation during the in vivo infection. Histopathological analysis demonstrated a higher number of neutrophils and macrophages, as well as increased necrosis area and edema, compared to wild-type mice. Moreover, infected Gal-3 KO mice presented an increased parasite burden in the footpads and draining lymph nodes [87]. Taken together, these data showed that Gal-3 is important to control the *Leishmania* infection, acting as a modulator of innate and adaptive immune responses during the *Leishmania* infection. However, *Leishmania* parasites possess mechanisms to evade and down-modulate Gal-3 activity, which contributes to promoting infection and replication in vivo. Therefore, the use of exogenous Gal-3 may contribute to controlling parasite burden, although further studies are necessary to prove this hypothesis.

**Table 2:** Main effects of galectins on Leishmaniasis experimental models.

Reference	Experimental Model	Main Results
[76]	Mice	Gal-1 knockout mice presented lower parasitism compared to wild-type mice, indicating infection by *L. donovani*.
[78]	Mice	Gal-3 deficiency resulted in reduced neutrophil infiltration in a model of cutaneous Leishmaniasis by infection with *L. major*
[79]	Mice	It was demonstrated that Gal-3 acted as a chemoattractant for neutrophils *in vitro*.
[80]	Mice	Gal-3 recognizes and binds to lipophosphoglycan from *L. major* but not from *L. donovani*. The binding of Gal-3 to *L. major* leads to truncated Gal-3
[81]	Human	Significant increase in circulating Gal-3 in samples from patients with PKDL as compared to health control
[83]	Mice	The lack of Gal-3 increases the frequency of TReg cells in the site of infection, as well as in the draining lymph nodes, in a mouse model of cutaneous Leishmaniasis by *L. major* infection
[86]	Mice	Gal-3 modulates T helper responses during *L. major* infection
[57]	Mice	Recongnition of *L. major* by Gal-9 through binding to the *L. major*-specific polygalactosyl epitope
[87]	Mice	During the experimental infection of mice with *L. amazonensis*, Gal-3 was involved in the control of parasite invasion, replication, recruitment of leukocytes, and the biogenesis of endocytic vesicles
[37]	Cell culture	Galectin isolated from the marine sponge *Chondrilla caribensis* presented anti-*Leishmania* activity against *L. infantum* promastigotes *in vitro*

Galectin (Gal); Post-Kala-azar dermal leishmaniasis (PKDL).

Finally, galectin isolated from the marine sponge *Chondrilla caribensis* was tested against *Leishmania*. The galectin presented anti-*Leishmania* activity against *L. infantum* promastigotes *in vitro*. The molecule induced apoptosis, following reactive oxygen species production, and caused impairment of the cell membrane of the parasites [37]. 

## 5. The Role of Galectins in Chagas Disease

**Figure 4:**
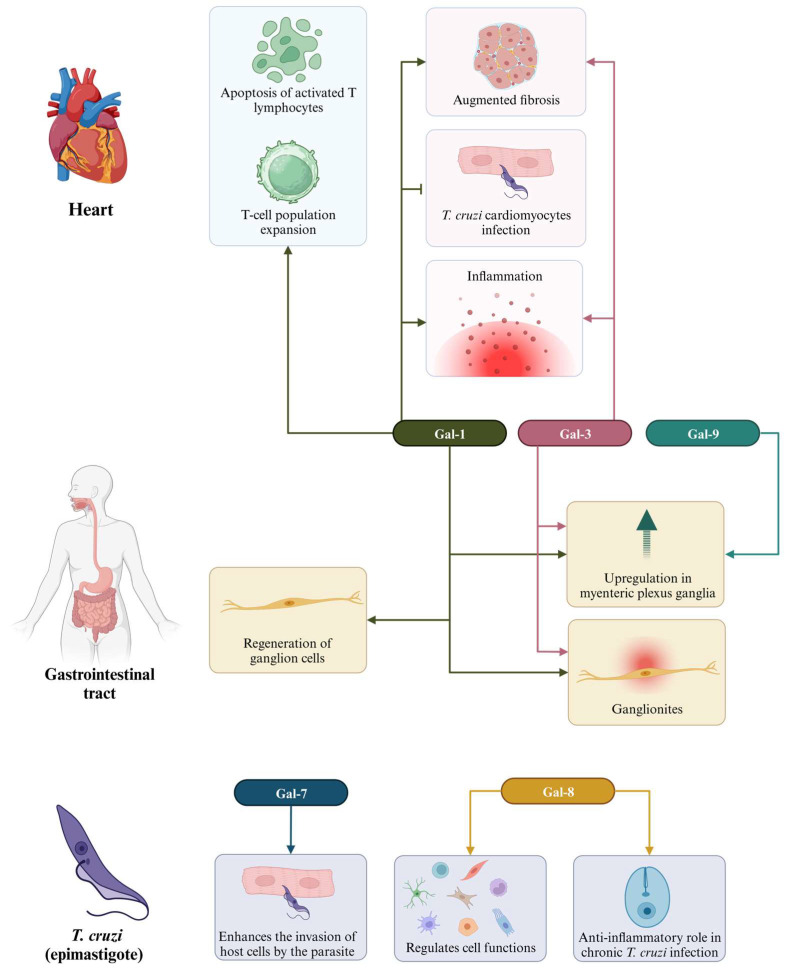
Different functions of galectins in Chagas disease. Different galectins are present in the heart, acting in the expansion of T-cells and activation of apoptosis by T lymphocytes (Gal-1), as well as in increasing fibrosis, inflammation, and prevention of *T. cruzi* infection (Gal-1 and Gal-3); in the gastrointestinal tract, we have the presence of Gal-1, Gal-3, and Gal-9, which act in the regeneration of ganglion cells; Gal-7 and Gal-8 assist in parasitic invasion into host cells, as well as in the regulation of cellular and anti-inflammatory functions, respectively, in *T. cruzi* epimastigotes.

The identification of biomarkers is a crucial step in the quest for strategies to diagnose and treat chronic Chagas cardiomyopathy (CCC). In this context, galectins (Gal) have emerged as subjects of extensive research related to cardiac dysfunction resulting from *T. cruzi* infection. CCC is characterized histologically by multifocal inflammation and fibrosis [88]. Several studies have demonstrated that galectins, particularly Gal-1 and Gal-3, play significant roles in the development of CCC (Table 3; Figure 4). This suggests that these molecules can serve as valuable biomarkers for both diagnosis and treatment. 

Gal-1, a glycan-binding protein, acts as an anti-inflammatory mediator and is highly expressed in activated T lymphocytes, tolerogenic dendritic cells, and inflammatory macrophages [89]. In the context of cardiac pathology resulting from *T. cruzi* infection, Gal-1 has been found to contribute to the pathophysiology of the disease. Although the parasite itself does not express Gal-1, patients with Chagas disease develop anti-Gal-1 antibodies during both the acute and chronic phases of the disease, elicited by antigens released from host cells [90]. Gal-1 is upregulated following infection and has been shown to play a role in disease progression by promoting apoptosis of activated T lymphocytes, expanding regulatory T cell populations, and limiting the clearance of the parasite [91,92]. Moreover, it acts as a cardioprotective factor by preventing *T. cruzi* infection in cardiomyocytes [93].

In a study developed by Benatar and colleagues [93], it was found that Gal-1 inhibits *T. cruzi* infection of cardiac cells. Moreover, parasite infection induces alterations in the surface glycophenotype of these cells, which restricts Gal-1 and potentially limits its inhibitory activity. Still in this investigation, in vivo experiments showed that mice lacking Gal-1 expression (Lgals1^−/−^) inoculated with *T. cruzi* (Tulahuen strain) had higher parasitemia and lower survival rates than their wild-type (WT) counterparts during the acute phase. Lgals1^−/−^ mice also exhibited a significantly greater density of parasitized cells and a lower inflammation score in the hearts of female mice and in the skeletal muscle of male mice compared with the WT controls. These findings suggest that modulation of Gal-1-glycan interactions in cardiac cells may play a role in parasite-induced heart injury [93].

On the other hand, the study conducted by Poncini et al. [91] found that Gal-1 functions as a negative regulator to limit host-protective immunity following intradermal infection with *T*. *cruzi* (RA strain) in acute infections. The researchers observed an early increase in Gal-1 expression concomitant with the upregulation of immune inhibitory mediators, including IL-10, TGF-β1, IDO, and programmed death ligand 2, in mice infected with *T. cruzi*. In comparison to their wild-type counterparts, Gal-1-deficient mice showed reduced mortality and a lower parasite load in muscle tissue during the acute phase of infection. The authors suggested that interruption of Gal-1-driven tolerogenic pathways during the acute phase of *T. cruzi* infection may promote parasite clearance and reduce the severity of the disease [91].

Zuñiga and colleagues [92] conducted a study to examine the expression and regulation of Galectin-1 (Gal-1) within the B-cell compartment, employing *T. cruzi* (Tulahuen strain) infection as a natural model for in vivo B-cell activation. They observed that Gal-1 was expressed on activated B cells from *T. cruzi*-infected mice, primarily localized in the cytosolic compartment. Additionally, the study found that, upon activation, B cells secreted Gal-1 into the extracellular environment. Furthermore, the researchers purified Gal-1 produced by activated B cells and discovered that it induced apoptosis (programmed cell death) specifically in T cells, not affecting B cells [92].

**Table 3:** Main effects of galectins on Chagas disease experimental models.

Reference	Experimental Model	Main Results
[90]	Human	Observed the occurrence of anti-Gal-1 autoAb in sera from patients in the acute and chronic stages of Chagas’ disease.
[92]	Mice	Gal-1 was expressed on activated B cells from *T. cruzi*-infected (Tulahuen strain) mice, and it induced apoptosis (programmed cell death) specifically in T cells.
[94]	Mice	Lack of Galectin-3 Prevents Cardiac Fibrosis and effective Immune Responses in a Murine model of *Trypanosoma cruzi* infection
[93]	Human and mice	Gal-1 (Lgals1^−/−^) exhibited higher parasitemia in the acute phase, diminished signs of inflammation in heart and skeletal muscle tissues, and lower survival rates compared to wild-type (WT) mice when intraperitoneally infected with the *T. cruzi* Tulahuen strain.
[91]	Mice	Alongside the heightened expression of immune inhibitory mediators and programmed death ligand 2, the infection of the *T. cruzi* RA strain triggered an early elevation of Gal-1 expression within living organisms. When compared to the wild-type (WT) mice, Gal-1-deficient (Lgals1^−/−^) mice demonstrated decreased mortality rates and lower parasite levels in their muscle tissue.
[95]	Cell culture	The passage states that galectins have a preference for binding to forms of a parasite that are present in the host (trypanosomatid trypomastigotes and amastigotes) compared to the non-infective epimastigote present in the intestinal tract of the vector. This is due to changes in glycosylation that occur during the metacyclogenesis and amastigogenesis processes.
[96]	Mice	Association of cardiac galectin-3 expression, myocarditis, and fibrosis in CCC
[97]	Mice and cell culture	Gal-3 is important to survival, migration, and immunomodulatory action, and Gal-3 knockdown MSC treatment does not reduce cardiac inflammation and fibrosis.
[98]	Human	There is no correlation between the degree of myocardial fibrosis and the concentration of Gal-3 in plasma samples from subjects with Chagas disease.
[99]	Human	Compared to non-chagasic patients, chagasic patients exhibited elevated expression of Gal-1, Gal-3, and Gal-9 in the myenteric plexus ganglia. The heightened presence of Gal-1 in the myenteric plexus ganglia of chagasic patients might play a role in the regeneration of ganglion cells, as Gal-1 is recognized for its ability to enhance axon plasticity and suppress macrophages.
[100]	Mice	DMS treatment reduces Gal-3 expression in the heart and serum of mice with chronic Chagas cardiomyopathy
[101]	Human	Higher levels of Gal-3 were significantly associated with severe forms of disease and a higher long-term mortality rate.
[102]	Mice	During the chronic phase, Gal-8-deficient mice exhibited widespread inflammation in the heart, skeletal muscle, and liver, leading to extensive fibrosis, independent of tissue parasite loads. Remarkably, there was a notable increase in the occurrence of neutrophils and macrophages as well.

*N*,*N*-dimethylsphingosine (DMS); Galectin (Gal); Mesenchymal stem cells (MSC); Autoantibodies (autoAB); chronic Chagas cardiomyopathy (CCC).

Beghini et al. [99] conducted a study aimed at assessing the immunohistochemical expression patterns of various galectins in the colon of chronic Chagas disease patients, comparing them to biopsied non-Chagas disease patients. Their investigation revealed heightened immunostaining levels for Gal-1, Gal-3, and Gal-9 within the myenteric plexus ganglia of individuals afflicted with Chagas disease. Furthermore, the authors proposed that the augmented expression of Gal-1 in the myenteric plexus ganglia of Chagas disease patients might contribute to the regeneration of ganglion cells, as Gal-1 is known to enhance axon plasticity while inhibiting macrophages. These findings suggest a potential association between Gal-1 and Gal-3 with ganglionitis in the Chagasic megacolon, offering novel insights into the pathogenesis of this ailment [99]. In contrast, the role of Gal-9 in Chagas disease remains unclear, despite its observed increase in expression in affected individuals.

Regarding Gal-3, it has been previously observed to have high expression levels in various cell types, including macrophages, T cells, and fibroblasts, whereby it exerts regulatory control over cell survival, proliferation, and collagen synthesis [96]. Notably, in vivo investigations have demonstrated that the inhibition of Gal-3 using *N*-acetyl-D-lactosamine leads to a reduction in cardiac fibrosis and inflammation in mice infected with *T. cruzi* [96]. Additionally, Gal-3 expression has been identified in regions of inflammation within human heart tissue samples from Chagas disease patients who underwent heart transplantation [101].

Souza and colleagues [97] demonstrated the significant role of Gal-3 in the survival, migration, and immunomodulatory functions of mesenchymal stromal cells (MSCs). MSC transplantation promoted a reduction of inflammation and fibrosis in a mouse model of CCC. This beneficial effect was associated with a reduction in the expression levels of key inflammatory markers such as CD45, TNFα, IL-1β, IL-6, IFNγ, and type I collagen. Gal-3 knockdown in transplanted MSC, however, led to impaired functionality of the cell therapy in mice with CCC [100]. These findings are consistent with those reported by Pineda et al. [95], who demonstrated that mice deficient in Gal-3 exhibited diminished fibrosis, reduced cell infiltration, and impaired immune responses in the context of *T. cruzi* infection. 

The levels of Galectin 3 are increased in both serum samples of mice [99] and humans [99] during the chronic phase of the disease, suggesting this molecule as a possible biomarker of disease evolution. Noya-Rabelo et al. [99] investigated the potential relationship between Gal-3 levels in the blood and myocardial fibrosis in Chagas disease patients. The authors found that Gal-3 levels did not present a significant correlation with myocardial fibrosis, indicating that it may not serve as an effective predictive biomarker for the progression to more severe disease forms. In contrast, Fernandes et al. [101] provided evidence suggesting that elevated Gal-3 levels are associated with the more severe manifestations of Chagas disease and increased long-term mortality rates.

The high levels of Gal-3 in the heart of CCC can be reduced after different treatments in the mouse model, as demonstrated by Vasconcelos and colleagues [100]. In their study, mice treated with the sphingosine kinase inhibitor *N*,*N*-dimethylsphingosine (DMS) exhibited a reduction in cardiac inflammation, fibrosis, and galectin-3 expression [100]. These results reinforce the idea that Gal-3 is an important biomarker in chronic Chagas disease. 

Other galectins identified in the context of Chagas disease are Gal-7 and Gal-8. Gal-7 has been identified as the only galectin with a significant bind to the epimastigote form of *T. cruzi*, the non-infective stage of the parasite’s life cycle [95]. Some authors have proposed the possibility that Gal-7 might act as one of the initial mediators facilitating the parasite’s entry into host cells [91,95]. Gal-8 represents another subtype of galectin with diverse functions, spanning both homeostatic and pathological processes. It exerts regulatory control over several cellular functions, including cytokine production, cellular adhesion, apoptosis, chemotaxis, endocytosis, differentiation, and migration across various cell types, including immune cells reviewed in [103]. In the context of chronic *T. cruzi* infection, Bertelli and colleagues [102] demonstrated that Gal-8 plays an anti-inflammatory role, facilitating the clearance of preaparesis and neutrophils by macrophages. The absence of Gal-8-dependent preaparesis clearance was associated with a widespread increase in inflammation in the heart, skeletal muscle, and liver, as well as extensive fibrosis, a phenomenon not correlated with parasite load within the tissues. The increased neutrophil count observed in Gal-8-deficient mice could be linked to the absence of Gal-8-dependent preaparesis and the compromised ability of macrophages to clear neutrophils, underscoring the importance of Gal-8 in regulating immune responses in the context of Chagas disease.

Lastly, we have Gal-9, which has been identified in the colons of chronic Chagas disease patients [99]. However, its precise role in the context of Chagas disease has not yet been fully elucidated. While Gal-9 is recognized as a valuable biomarker for assessing the severity of various other diseases, including autoimmune disorders, viral infections, cancer, and parasitic invasions, its specific function and significance in Chagas disease remain to be determined. Further investigations are needed to unravel the contributions and implications of Gal-9 in the pathogenesis and progression of Chagas disease.

Indeed, Chagas disease remains without a definitive therapy, making the identification of biomarkers for its progression control of utmost importance. Galectins, with a particular focus on Gal-1 and Gal-3, have emerged as noteworthy proteins with diverse roles in this neglected tropical disease. These findings offer fresh perspectives on its pathogenesis and hold the promise of serving as potential therapeutic targets for the development of new treatments for individuals afflicted with Chagas disease.

## 6. Concluding Remarks

First, it is crucial to acknowledge, in alignment with the inherent nature of literature reviews, that a certain degree of subjectivity is involved in the application of filtering criteria, and alternative choices may be made by other researchers. It is possible that relevant articles are published in journals not covered by the databases we searched.

In the context of the evaluated parasitic diseases, we found that galectins play a dynamic role across multiple pathways encompassing pathogen recognition, parasite invasion and replication, and the modulation of both innate and adaptive immune responses. Notably, galectins 2, 3, and 9 emerge as key players in malaria, while galectins 1 and 3 are central in Chagas disease and Leishmaniasis. This multifaceted engagement positions certain galectins as possible biomarkers of disease severity, as exemplified by the potential utilization of galectin-9 as a biomarker for severe malaria. Nevertheless, the prognostic significance of galectins needs better validation through investigations involving a larger cohort of patients.

Furthermore, owing to their pivotal roles in the pathogenesis of these parasitic diseases, some galectins present as promising targets for the advancement of novel therapeutic interventions. Galectin-3, implicated in the promotion of inflammation and fibrosis in chronic Chagasic cardiomyopathy, is actively explored as a therapeutic target in the quest for the discovery of new drugs. Despite the notable potential of galectins for diagnostic and therapeutic applications in the realm of neglected diseases, it is imperative to undertake further experimentation, specifically employing murine models devoid of galectins in relevant tissues. Such investigations are essential to garner a more comprehensive understanding of the distinct roles that galectins play in the context of different clinical manifestations of Chagas diseases, Leishmaniasis, and Malaria. Additionally, the integration of glycan array technologies holds promise for elucidating the biochemical determinants driving parasite-galectin interactions. Moreover, the validation of diagnostic and therapeutic applications of galectins in the context of parasitic diseases, as exposed in this review, necessitates in-depth in vitro and ex vivo assays utilizing human cells. Additionally, further investigations are imperative to identify and characterize galectins expressed by protozoan parasites. Finally, it is essential to conduct clinical studies to comprehensively explore the therapeutic potential of galectins for these neglected tropical diseases.
